# Supplementary material for: Effects of Therapeutic Hypothermia on Macrophages in Mouse Cochlea Explants
Source: Int J Mol Sci. 2023 May 16;24(10):8850. doi: 10.3390/ijms24108850 (PMC10218374; doi:10.3390/ijms24108850)
Supplement: Supplementary file 1 [file ijms-24-08850-s001.zip › ijms-2331006-supplementary.pdf]

Supplementary Material

Supplement Table S1: Composition of Cochlea Culture Media

**Component**	**Final Concentration**	**Vol. 10 ml (one vessel)**
BDNF	10ng/ml	100µl
NT-3	5ng/ml	50µl
Neurobasal Media (Gibco, 21103-049)	ready to use concentration	9,35 ml
L-Glutamine (200 mM)	5 mM	250 µl
HEPES-Buffer (1 M, PAA, S11-001)	10 mM	100 µl
Penicillin G (20000 units)	100 units/ml	100 µl
B27 Supplement (Invitrogen, 17504-044 50x stock)	1x	200 µl
NaOH (for pH adjustment to 7,4)	1 M	30 µl

Supplement Table S2: Used primary and secondary antibodies with host, dilution and catalogue number.

**Antibody**	**Type**	**Host**	**Dilution**	**Producer, Catalogue Number**
IBA1	Monoclonal	Rabbit	1:100	Abcam, ab178847
F4/80	Monoclonal	Rat	1:50	Abcam, ab6640
CD45	Monoclonal	Goat	1:100	R&D System, AF114
CD163	Monoclonal	Rabbit	1:200	Abcam, ab182422
Alexa Fluoro Donkey Anti Rabbit 594	Polyclonal	Rabbit	1:200	Invitrogen, A21207
Alexa Fluoro Donkey Anti Goat 546	Polyclonal	Goat	1:200	Invitrogen, A11056
Alexa Fluoro Donkey Anti Rat 488	Polyclonal	Rat	1:200	Invitrogen, A21208
